# Cesarean section and risk of allergies in Ecuadorian children: A cross‐sectional study

**DOI:** 10.1002/iid3.368

**Published:** 2020-10-30

**Authors:** Amélie Gorris, Gabriela Bustamante, Katharina A. Mayer, Tamar Kinaciyan, Gerhard J. Zlabinger

**Affiliations:** ^1^ School of Medicine Universidad San Francisco de Quito Quito Ecuador; ^2^ Institute of Immunology, Center of Pathophysiology, Infectiology and Immunology Medical University of Vienna Vienna Austria; ^3^ Department of Dermatology Medical University of Vienna Vienna Austria; ^4^ Program in Health Disparities Research University of Minnesota Minneapolis Minnesota USA

**Keywords:** asthma, atopic dermatitis, environment and hygiene hypothesis, food allergy, rhinitis

## Abstract

**Background:**

Studies have shown an association between cesarean section (CS) and increased prevalence of childhood allergic diseases. While these observations have been consistent in industrialized countries, evidence from developing countries is limited.

**Objective:**

To assess the association between the mode of delivery and allergic diseases in children aged 3–12 years in Quito, Ecuador.

**Methods:**

In this cross‐sectional study, parents were surveyed using an anonymous, standardized questionnaire from the International Study of Asthma and Allergies in Childhood project to assess the presence of asthma, allergic rhinitis, atopic dermatitis, and food allergies in their children. The children's age, sex, birthplace, delivery mode (CS/vaginal), socioeconomic status, and ethnicity were recorded. Other parameters included gestational age, breastfeeding, smoking status during pregnancy, and parental allergic diseases.

**Results:**

After adjusting for confounding factors, children delivered via CS were found to have a higher risk of wheezing (odds ratio [OR] = 4.12, 95% confidence interval [CI]: 1.43–11.89), physician‐diagnosed asthma (OR = 24.06; 95% CI: 1.98–292.3), and pimples, or eczema with the itching for 6 months (OR = 2.65; 95% CI: 1.06–6.61) than children delivered vaginally. No association was found between the delivery mode and rhinitis or food allergies. After stratifying by socioeconomic status, CS was only associated with allergic disorders in children of medium/high socioeconomic backgrounds.

**Conclusions:**

As seen in industrialized settings, children born by CS in nonindustrialized countries have an increased risk of developing allergic disorders including asthma and dermatitis, compared to those delivered vaginally.

## Graphical abstract

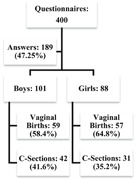

**Abbreviations**
CI: confidence interval
CS: cesarean section
ISAAC: International Study of Asthma and Allergies in Childhood
OR: odds ratio
WHO: World Health Organization

## 1 INTRODUCTION

The prevalence of allergic diseases and asthma is increasing worldwide.^1^ Globally, 300 million people suffer from asthma, approximately 200–250 million people have food allergies and 400 million people have rhinitis.^1^, ^2^ According to the World Health Organization (WHO), the number of patients with asthma is expected to increase to 400 million by 2025.^2^ This requires even greater awareness of the underlying causes. A recent study by Olin et al.^3^ investigated the impacts of early life (both environmental and genetic), such as mode of delivery, gestational age, length of hospital stay, nutritional status of the mother and newborn, exposure to infectious agents, microbiome colonization, and antibiotic use. The study established that all of these factors could shape immune reactivity and also play a part in determining the occurrence of allergies.^3^ According to the hygiene hypothesis, the increasing rate of allergic diseases is related to environmental factors such as improvements in hygiene.^4^, ^5^ Therefore, allergies are expected to be more prevalent in industrialized countries. It is quite remarkable that while more than half of Latin American countries report a prevalence rate greater than 15% for childhood asthma,^6^ the prevalence rates of asthma are approximately 9% among children^6^, ^7^ in Europe and the United States. For this reason, other factors may play an important role in the induction of allergic diseases.

In recent decades, the incidence of delivery by cesarean section (CS) has increased substantially.^8^ Several recent studies have suggested that delivery by CS is associated with increased incidence of allergic disease in children in industrialized countries; however, data on the risk of allergic disorders after CS in developing countries is very limited.^9^, ^10^ While the prevalence of asthma, allergic diseases, and risk factors for allergic disorders in Latin American populations have been assessed,^6^, ^11^, ^12^, ^13^, ^14^ and special interest has been paid to the increasing rates of CS in Ecuador,^15^ there have not been enough targeted studies investigating a link between the mode of delivery and the development of allergic diseases. Based on data from 121 countries, the global average CS rate between 1990 and 2014 increased by 12.4% (from 6.7% to 19.1%).^8^ The largest increases were recorded in Latin America and the Caribbean (from 19.4% to 42.2%).^8^ The CS rate in Ecuador reached almost 43% in 2014.^16^, ^17^ This is far higher than the index put forth by WHO, which proposes an ideal CS rate of 10%–15%.^17^

Given both, the high prevalence of childhood allergies and the striking rate of CS, we hypothesized an association of these two events and carried out a cross‐sectional study. In this analysis, we evaluated the association between the mode of delivery and allergic diseases among Ecuadorian children.

## 2 METHODS

### 2.1 Study cohort

This analysis was carried out within the scope of a cross‐sectional study that surveyed the parents of 400 children aged 3–12 years living in Quito, Ecuador, from 2014 to 2015; 300 children attended a private school and 100 attended a public school. Considering the fact that private schools are mostly visited by children with socioeconomic high‐/medium‐income parents and public school by children with socioeconomic lower income parents, this strategy was chosen to pay attention to the impact of the large socioeconomic and social differences that prevail in Ecuador. Informed consent was obtained from all participants. A standardized questionnaire from the International Study of Asthma and Allergies in Childhood (ISAAC) project was used for data collection.^18^ During the survey, closed questions were asked about the mode of delivery (CS or vaginal) and whether an allergy had been diagnosed by a physician. In addition, four clinical manifestations were assessed: asthma, allergic rhinitis, atopic dermatitis, and food allergy (both physician‐diagnosed and self‐reported). These questions were supplemented with specific inquiries regarding the symptoms of each disease with standard ISAAC definitions, such as asthma (wheezing or whistling in the chest), current rhinoconjunctivitis (a problem with sneezing or a runny or blocked nose without a cold or flu, accompanied by itchy watery eyes), and eczema (itchy rash at any time affecting folds of the bows, behind the knees, in front of the ankles, under the buttocks or around the neck, ears, or eyes).

To account for potential confounders, we collected information on the following additional variables: age of the child, sex, socioeconomic status, ethnicity, place of birth, premature birth (delivery at a gestational age less than 37 weeks), breastfeeding (whether a child was exclusively breastfed in the first 6 months), diagnosed maternal or paternal allergy, any smoke exposure at home, and daycare attendance in the first year of life (Table 1). Socioeconomic status was established via specific questions on income, based on the minimum wage in Ecuador (354 USD/month in 2015).^19^ These groups were further divided into two subgroups referred as lower (≤$354–500) and medium to high (>$500) in monthly income.

**Table 1:** Baseline characteristics of the study population

Variables	Total number	Number vaginal delivery (%)	Number cesarean section delivery (%)	*p*
Sex				
Female	88	57 (64.8)	31 (35.2)	.37
Male	101	59 (58.4)	42 (41.6)	
Age	8.24 ± 2.69	8.16 ± 2.60	8.38 ± 2.86	.59
Ethnicity				
Mestizo (mixed race)	146	87 (59.6)	59 (40.4)	.30
White	17	9 (52.9)	8 (47.1)	
Indigenous	13	10 (76.9)	3 (23.1)	
Afro‐Ecuadorean	4	4 (100)	0 (0.00)	
Other	7	5 (71.4)	2 (28.6)	
Socioeconomic status				≤**.001**
Medium/high	94	38 (40.4)	56 (59.6)	
Low	95	78 (82.1)	17 (17.9)	
Born in Quito				
Yes	141	85 (60.3)	56 (39.7)	.74
No	38	24 (63.2)	14 (36.8)	
Premature birth				
Yes	14	5 (35.7)	9 (64.3)	**.03**
No	160	104 (65.0)	56 (35.0)	
Breastfeeding				
Yes	177	110 (62.1)	67 (37.9)	.44
No	10	5 (50.0)	5 (50.0)	
Breastfeeding duration				
Did not breastfeed	10	5 (50.0)	5 (50.0)	.66
Less than 6 months	31	18 (58.1)	13 (41.9)	
6 to 12 months	69	45 (65.2)	24 (34.8)	
13 to 24 months	50	28 (56.0)	22 (44.0)	
More than 24 months	26	18 (69.2)	8 (30.8)	
Daycare attendance				
Yes	100	52 (52.0)	48 (48.0)	**.005**
No	83	60 (72.3)	23 (27.7)	
Mother with diagnosed allergy				
Yes	33	16 (48.5)	17 (51.5)	.09
No	151	97 (64.2)	54 (35.8)	
Father with diagnosed allergy				
Yes	27	10 (37.0)	17 (63.0)	**.008**
No	151	98 (64.9)	53 (35.1)	
Mother smoked				
In pregnancy	16	7 (43.8)	9 (56.2)	.06
First year of child	3	2 (66.7)	1 (33.3)	
At some point of life	3	0 (0.00)	3 (100)	
Never	165	106 (64.2)	59 (35.8)	
Any smokers at home				
Yes	39	25 (64.1)	14 (35.9)	.77
No	146	90 (61.6)	56 (38.4)	

Abbreviations: *p* values are calculated using the *χ* ^2^ test for nominal variables and the Student *t* test for continuous variables. Statistically significant values are presented in bold. Total number of collected questionnaires: 189.

**Figure 1:**
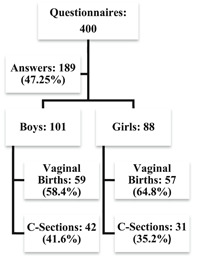
Study enrollment flowchart. Parents of 400 children did participate in the present study and answered 189 questionnaires. The response rate was 47.25%.

### 2.2 Statistical analysis

First, we evaluated the relationship between demographic and health‐related covariates in the delivery mode. Nominal variables were assessed using a *χ* ^2^ test and continuous variables were assessed using a Student's *t* test. Associations with *p* < .05 were considered statistically significant. To investigate the link between the delivery mode and allergic diseases, binary logistic regression analysis was performed to estimate the crude odds ratio (OR) and 95% confidence interval (CI). ORs and 95% CIs were calculated using logistic regression analysis. The adjusted OR and 95% CI were calculated using a multivariate model that included factors from Table 1 that had a *p* < .05 in the binary logistic model. Specifically, the adjusted estimates accounted for sex, age, socioeconomic status, premature birth, daycare attendance, father with allergy, and any smoke exposure at home. Statistical analyses were performed using SPSS version 20 (IBM Corp.).

## 3 RESULTS

We invited the parents of 400 children to participate in the present study and received 189 responses to the questionnaires. The response rate was 47.25% (Figure 1), which is in line with the findings of Yu and Cooper,^20^ whose study yielded a response rate of 50%. The mean age (and *SD*) of the children included in our study was 8.2 ± 2.7 years. The characteristics of the children that may influence allergies^21^ such as gender, age, ethnicity, socioeconomic status, city of birth, premature birth, lactation, daycare attendance, exposure to smoke, and parents with allergies are summarized in Table 1 for the entire sample and by mode of delivery. Only children were included in our study, as most allergies develop early in life.^21^

Among the 189 children included, 116 (61.4%) were delivered vaginally and 73 (38.6%) via CS. This is in line with public data collected in Ecuador in 2014, which showed a CS rate of 43%, indicating that our study utilized accurate sample. Of the children delivered via CS, 76.7% had a medium to high socioeconomic background. Furthermore, of the 94 children with a medium to high socioeconomic background, 59.6% were delivered via CS compared with 17.9% among those with a low socioeconomic background (*p* < .001). This supported our hypothesis that CS is more common among mothers with higher socioeconomic status, as reported by Ortiz‐Prado et al.^15^ and Jahnke et al.^15^, ^22^

As shown in Table 1, we found that the following factors were associated with the mode of delivery. In the premature birth group, 64.3% of children were delivered via CS, whereas only 35% of the children born at term were delivered via CS (*p* = .03). This finding reflects the fact that CS is more commonly performed in premature births. We found that the prevalence of paternal history of allergy was associated with cesarean delivery (*p* = .02). We hypothesized that this could be due to a genetic predisposition, but furthermore after adjusting for allergic conditions, we found that this suspected genetic factor had no relation to the mode of delivery, leaving this point inconclusive. Among children who had attended daycare, 48% were delivered via CS, whereas among children who did not attend daycare, only 27.7% were delivered via CS (*p* = .005). It could be hypothesized that children who attend nursery are more likely to be delivered via CS; however, further analysis is needed. There were no significant differences in gender, ethnicity, breastfeeding, or whether the children were exposed to smoke at home (Table 1).

To establish the risk of allergies in children delivered via CS, we first calculated a crude OR for asthma, rhinitis, atopic dermatitis, and food allergy. In our study, delivery via CS was found to significantly increase the risk of physician‐diagnosed asthma, wheezing, physician‐diagnosed eczema, pimples, or eczema with itching over 6 months, without adjusting for confounding variables (Table 2). However, delivery via CS showed no association with physician‐diagnosed allergic rhinitis and food allergies. To further evaluate these associations, we assessed whether the mode of delivery was clearly associated with an increased risk of allergic diseases in offspring, and adjusted for confounding factors including sex, age, socioeconomic status, premature birth, exclusive breastfeeding, daycare attendance, father with allergy, born in Quito, and smoke exposure at home. After adjusting for these covariates, we found that delivery via CS increased the risk of asthma by a factor of 24.1 (95% CI: 1.98–292.3), wheezing by 4.12 (95% CI: 1.43–11.89), and pimples or eczema with itching for 6 months by 2.65 (95% CI: 1.06–6.61; Table 2). No significant results were obtained for eczema after adjusting for confounding factors. The main factor influencing the development of diagnosed rhinitis was daycare attendance (OR = 3.33; 95% CI: 1.35–8.33). Socioeconomic status was significantly related to all allergic manifestations (Table 2; *p* < .001).

**Table 2:** Association between the mode of delivery and allergic diseases

Confounding variables	Crude OR (95% CI)	Adj. OR (95% CI)	Crude OR (95% CI)	Adj. OR 1 (95% CI)
Asthma	Physician‐diagnosed asthma		Wheezing	
Type of delivery (cesarean vs. vaginal)	**20.2 (2.55–160.2)** *	**24.1 (1.98–292.3)** *	**3.76 (1.63–8.67)** *	**4.12 (1.43–11.89)** *
Age (y)	1.12 (0.81–1.02)		1.06 (0.92–1.23)	
Gender (Female vs. Male)	0.81 (0.25–2.65)		0.66 (0.29–1.49)	
Socioeconomic status (medium/high vs. low)	**5.61 (1.15–25.0)** *		**2.56 (1.11–5.88)** *	
Premature birth (yes vs. no)	1.14 (0.13–10.0)		1.39 (0.36–5.26)	
Breastfeeding minimal (yes vs. no)	0.60 (0.07–5.26)		0.73 (0.15–3.57)	
Daycare attendance (yes vs. no)	0.82 (0.25–2.63)		1.10 (0.49–2.50)	
Smoker at home (yes vs. no)	2.00 (0.57–7.14)		1.01 (0.38–2.70)	
Born in Quito (yes vs. no)	3.11 (0.39–25.0)		1.35 (0.47–3.83)	
Father with diagnosed allergy	0.32 (0.92–1.12)		0.42 (0.15–1.15)	
Rhinitis	Physician‐diagnosed rhinitis		Runny or blocked nose	
Type of delivery (cesarean vs. vaginal)	1.84 (0.85–4.07)	0.99 (0.37–2.62)	2.18 (1.19–3.98)	1.41 (0.65–3.08)
Age (y)	1.02 (0.88–1.19)		1.02 (0.91–1.14)	
Gender (Female vs. Male)	1.04 (0.47–2.27)		1.05 (0.58–1.89)	
Socioeconomic status (medium/high vs. low)	**6.25 (2.33–16.7)** *		**2.22 (1.22–4.17)** *	
Premature birth (yes vs. no)	0.88 (0.18–4.17)		2.13 (0.70–6.25)	
Breastfeeding minimal (yes vs. no)	0.43 (0.11–1.79)		0.95 (0.26–3.45)	
Daycare attendance (yes vs. no)	**3.33 (1.35–8.33)** *		1.37 (0.75–2.50)	
Smoker at home (yes vs. no)	1.47 (0.59–3.57)		1.12 (0.55–2.33)	
Born in Quito (yes vs. no)	0.57 (0.24–1.40)		1.06 (0.51–2.22)	
Father with diagnosed allergy	.30 (0.12–0.76)		0.34 (0.15–0.76)	
Eczema	Physician‐diagnosed eczema		Pimples or eczema with itching for 6 months	
Type of delivery (cesarean vs. vaginal)	**2.47 (1.09 – 5.60)** *	1.14 (0.42–3.11)	**2.64 (1.31–5.29)** *	**2.65 (1.06–6.61)** *
Age (y)	1.01 (0.88–1.13)		0.90 (0.78–1.05)	
Gender (Female vs. Male)	1.31 (0.66 – 2.60)		0.86 (0.38–1.94)	
Socioeconomic status (medium/high vs. low)	1.72 (0.85 – 3.45)		**8.33 (2.63–25.0)** *	
Premature birth (yes vs. no)	2.08 (0.65 – 6.67)		0.42 (0.05–3.33)	
Breastfeeding minimal (yes vs. no)	1.23 (0.25–5.88)		1.47 (0.18–12.5)	
Daycare attendance (yes vs. no)	1.33 (0.66–2.70)		1.41 (0.62–3.23)	
Smoker at home (yes vs. no)	1.14 (0.50–2.56)		1.08 (0.40–2.86)	
Born in Quito (yes vs. no)	1.34 (0.47–3.80)		0.67 (0.30–1.51)	
Father with dg. allergy	0.37 (1.14–0.98)		0.53 (0.23–1.24)	
Food allergy	Physician‐diagnosed food allergy		Food allergy in any moment of life	
Type of delivery (cesarean vs. vaginal)	2.62 (0.89–7.70)	1.73 (0.43–7.00)	2.30 (0.95–5.55)	1.48 (0.48–4.64)
Age (y)	0.94 (0.78–1.15)		1.08 (0.91–1.27)	
Gender (Female vs. Male)	0.56 (0.18–1.69)		0.87 (0.36–2.09)	
Socioeconomic status (medium/high vs. low)	**4.55 (1.20–16.7)** *		**4.17 (1.52–12.5)** *	
Premature birth (yes vs. no)	3.70 (0.59–14.3)		3.13 (0.89–11.1)	
Breastfeeding minimal (yes vs. no)	O/A		O/A	
Daycare attendance (yes vs. no)	1.28 (0.44–3.70)		1.67 (0.67–1.12)	
Smoker at home (yes vs. no)	2.00 (0.64–6.25)		2.27 (0.88–5.88)	
Born in Quito (yes vs. no)	0.47 (0.15–1.50)		0.70 (0.25–2.00)	
Father with diagnosed allergy	0.22 (0.07–0.67)		0.31 (0.11–0.84)	

*Note*: ORs and 95% CIs are calculated using logistic regression analysis. The adjusted OR and 95% CI are calculated using a multivariate model that included factors identified in Table 1: sex, age, socioeconomic status, premature birth, exclusive breastfeeding, daycare attendance, father with allergy, born in Quito, and smoke exposure at home. Statistically significant values are presented in bold.

Abbreviations: CI, confidence interval; F, female; M, male; OR, odds ratio.

* *p* ≤ .05.

To assess whether socioeconomic status modified the association of delivery mode with allergies, we conducted a stratified analysis by comparing the likelihood of the development of allergic diseases in children according to socioeconomic status (high vs. low) after adjusting other confounding factors except life‐style in more hygienic setting. Socioeconomic status was significantly related to all allergic manifestations (Table 2; *p* < .001). Furthermore, children delivered via CS were more susceptible to asthma (Table 3; OR = 7.04; 95% CI: 0.85–58.2) than those who were delivered vaginally in the population with high socioeconomic status. CS increased the risk of wheezing (OR = 3.49; 95% CI: 1.06–11.4) and pimples or eczema with itching for 6 months (OR = 4.08; 95% CI: 1.37–12.1). This is congruent with the results regarding a higher CS rate in this group. The mode of delivery showed no significant association with rhinitis (OR = 1.09; 95% CI: 0.43–2.80) or food allergies (OR = 2.23; 95% CI: 0.56–8.86) in high socioeconomic status (Table 3). In the group with lower socioeconomic status, we found no significant relationship between the mode of delivery and allergy manifestations (Table 3). However, this may also be due to the small number of children delivered via CS in this group.

**Table 3:** Association between mode of delivery and allergic diseases as depending on socioeconomic status

	Medium/high socioeconomic status			Low socioeconomic status		
Type of birth		OR simple (IC 95%)		OR simple (IC 95%)		
	Physician‐diagnosed asthma			Physician‐diagnosed asthma		
	With	Without	**7.04 (0.85–58.2)** *	With	Without	N/A
Vaginal	1 (2.7%)	36 (97.3%)		0 (0.0%)	76 (100%)	
Cesarean	9 (16.3%)	46 (83.7%)		2 (11.7%)	15 (88.3%)	
	Wheezing			Wheezing		
	With	Without	**3.49 (1.06–11.4)** *	With	Without	2.53 (0.56–11.4)
Vaginal	4 (10.5%)	34 (89.5%)		6 (7.8%)	71 (92.2%)	
Cesarean	16 (29.1%)	39 (70.9%)		3 (17.6%)	14 (82.3%)	
	Physician‐diagnosed rhinitis			Physician‐diagnosed rhinitis		
	With	Without	1.09 (0.43–2.80)	With	Without	N/A
Vaginal	10 (27.0%)	27 (73%)		5 (6.6%)	71 (94.4%)	
Cesarean	15 (28.8%)	37 (71.2%)		0 (0.0%)	16 (100%)	
	Runny or blocked nose			Runny or blocked nose		
Vaginal	With	Without	1.48 (0.64–3.39)	With	Without	1.02 (0.32–3.25)
	16 (42.1%)	22 (57.9%)		22 (28.9%)	54 (71.1%)	
Cesarean	29 (51.8%)	27 (48.2%)		5 (29.4%)	12 (70.6%)	
	Physician‐diagnosed eczema			Physician‐diagnosed eczema		
Vaginal	With	Without	1.12 (0.43–2.94)	With	Without	1.66 (0.16–17.1)
	9 (25.0%)	27 (75%)		3 (3.8%)	75 (96.2%)	
Cesarean	15 (27.3%)	40 (59.7%)		1 (6.25%)	15 (93.75%)	
	Pimples or wheals with itching for 6 months			Pimples or wheals with itching for 6 months		
Vaginal	With	Without	**4.08 (1.37–12.1)** *	With	Without	1.12 (0.28–4.52)
	5 (13.2%)	33 (86.8%)		14 (18.2%)	63 (81.8%)	
Cesarean	21 (38.2%)	34 (61.8%)		3 (20%)	12 (80%)	
	Physician‐diagnosed food allergy			Physician‐diagnosed food allergy		
Vaginal	With	Without	2.23 (0.56–8.86)	With	Without	N/A
	3 (7.9%)	35 (92.1%)		3 (3.8%)	75 (96.2%)	
Cesarean	9 (16.1%)	47 (83.9%)		0 (0%)	16 (100%)	
	Food allergy in any moment of life			Food allergy in any moment of life		
Vaginal	With	Without	1.99 (0.65–6.16)	With	Without	N/A
	5 (13.2%)	33 (86.8%)		5 (6.4%)	73 (93.6%)	
Cesarean	13 (23.2%)	43 (76.8%)		0 (0%)	17 (100%)	

*Note*: ORs are based on multivariate logistic regression analysis adjusted for the following factors: sex, age, socioeconomic status, premature birth, exclusive breastfeeding, daycare attendance, father with allergy, born in Quito, and smoke exposure at home. Statistically significant values are presented in bold.

Abbreviations: CI, confidence interval; N/A, not assessed since frequency 0 appeared in this category; OR, odds ratio.

* *p* ≤ .05.

## 4 DISCUSSION

Latin America recorded the highest increase in CS rate between 1990 and 2014. Ecuador, in particular, presented one of the highest rates of increase, driven by a small baseline CS rate and rapid economic development.^15^, ^22^ As the healthcare system improved and disposable incomes increased, more mothers opted for CS as a mode of delivery. Ortiz‐Prado et al.^22^ investigated CS rates in Ecuador and found that the overall national CS rate in the private healthcare system was double that of the public healthcare system. Pilot studies in Ecuador have demonstrated a relationship between CS and inflammatory diseases, which may predispose to future infections.^23^, ^24^

Therefore, it was appropriate to study the association between the delivery mode and allergic diseases in a nonindustrialized country in this setting by conducting a cross‐sectional study of children aged 3–12 years living in Ecuador. The relationship between the current “allergy epidemic” and key environmental factors such as environmental pollution, lifestyle, nutrition, and bacterial contact in early childhood, has been investigated in the Latin American population,^12^, ^13^, ^14^, ^25^ but limited evidence exists on how the prevalence of allergic diseases is associated with the high rate of CS and the impact of socioeconomic background.^15^, ^23^ We found that delivery via CS strongly increased the risk of developing asthma and wheezing (OR = 20.2 and 3.76, respectively), independently of industrialization, which was also evident after adjusting for confounding factors such as sex, premature birth exclusive breastfeeding, nursery attendance, parents' allergic status, and exposure to tobacco smoke (OR = 24.1 and 4.12, respectively).

Understanding the dynamics implicated in initial neonatal immune interactions could provide critical clues to the prevention of lifelong diseases. Neonates present a significant defect in T and B cell signaling, as described in a recent study conducted by Olin et al.^3^ that assessed the immune cell population in 100 newborns during the first 3 months of life. The authors found that during this critical window of time, the development of B cells, natural killer cells, and dendritic cells is critical, as these cells reach adult‐like patterns during this period.^3^ This suggests that environmental factors imprinted in these cells during the first 3 months of life could have lifelong impacts on the immune system and also implies that reduced levels of anergized innate immune cells, compromised cellular extravasation, Th2‐inflammatory biases, and delayed generation of antibodies may hinder the immune response upon infection.^3^ Most studies report that CS causes a change in the normal composition of the bacterial flora as well as an increased vulnerability and growth of unfavorable microorganisms.^26^, ^27^ A change in the normal gut flora might, therefore, affect the response of an organism to future infections and influence subsequent immune reactions to harmless non‐self antigens, including allergens.^21^, ^28^, ^29^ Olin et al. also profiled early dysbiosis in gut microbiome using fecal samples obtained at Weeks 1, 4, and 12 of life, and found that children with early gut dysbiosis had higher levels of microbiome dysregulation.^3^ Further investigations into the possible differences in immune development are needed.

We show here that delivery via CS strongly increases the risk of developing asthma more than 20‐fold. Several epidemiological studies have investigated the risk of developing allergic disease among children delivered via CS in highly industrialized countries. As such, a meta‐analysis conducted in 2008 found a 20% increase in the risk of asthma among subjects who were delivered via CS compared with those delivered vaginally.^30^ In a prospective study on asthma conducted in Copenhagen, children delivered via CS had a two‐fold risk of developing an asthmatic disease compared with children delivered vaginally.^31^

However, the authors found a heterogeneity in the various regions investigated in their study. In line with this, the prevalence of allergic disorders and their relationship to delivery via CS has to be assessed separately in each specific region (i.e., according to geographic region, highly industrialized versus developing countries, and socioeconomic differences). It has been hypothesized that early infections with helminths may affect the immune response of T‐helper type 2 cells, which can influence further development of allergic diseases.^32^ In a recent study conducted in Ecuador, maternal and childhood infections with helminths up to the age of 3 years had contrasting effects on the risks of wheezing and asthma (with maternal geohelminths increasing the risk and childhood infections decreasing the risk).^33^ However, the study did not assess the association with CS, which might be one of the confounding factors that influenced the contrasting results. The increased risk that we found in our study is far higher than is reported in the aforementioned studies conducted in highly industrialized countries, this fact might be due to specific environmental factors. A study conducted at the University of Melbourne proposed that people living near the equator have a higher risk of developing allergic disorders, probably because of an increased variation in allergens.^34^ A disturbance in the microbiome after CS, combined with a higher exposure to allergens, and a defective immune response could account for the higher risk of allergic disorders.

Conflicting evidence exists regarding the occurrence of allergic rhinitis after CS delivery. Pistiner et al.^35^ proposed a link between CS and allergic rhinitis among offspring when looking at parents with a history of atopic disease. In line with the results of the present study, a retrospective study conducted in Denmark failed to establish a link between CS and the development of rhinitis.^36^ However, it has to be considered that differences exist between children from different countries and socioeconomic backgrounds. The subgroup analyses of a recent prospective study conducted by Richards et al.^37^ revealed that CS without exposure to the maternal microbiome yielded higher risk ratios than CS involving exposure to the maternal microbiota. It could be hypothesized that another form of immune response underlies the pathophysiology of rhinitis, suggesting the need for further investigation.

In this study, we found a significant association between CS and atopic dermatitis (OR = 2.63). In a recent study conducted by Rehbinder et al.,^38^ CS was associated with a higher risk of atopic dermatitis. Interestingly, the authors found that these allergic responses were greater during the first 3 months of life, as reported by Olin et al.,^3^ who hypothesized that the first 3 months represent a critical but unappreciated window of immunological pressure in which environmental factors shape the immune system. Rehbinder et al.^38^ described dry skin as the major risk factor for atopic eczema, which seemed to be dysregulated among children delivered via CS. This may be explained by the shorter exposure time to the maternal immune system and Th2 cytokines, lower levels of Immunoglobulin E, and a different composition of early gut and skin microbiome.^38^ However, studies conducted in Europe with small sample sizes (Gothenburg [*n* = 116], London [*n* = 108], and Rome [*n* = 100]) have been unable to establish a similar relationship.^39^ In the present study, the link between delivery via CS and the occurrence of food allergies could not be established. Although we did not find an association between food allergies and CS, we were able to delineate the influence of socioeconomic status on the development of food allergies since we found that children with middle to higher socioeconomic status are more prone to food allergies than those with lower socioeconomic status. A recent study conducted by Levin et al.^40^ in Africa showed that environmental factors influence the occurrence of different food allergies in children living in urban and rural areas. Other studies also reported significant differences between infants delivered vaginally and those delivered via CS with regard to clinical features and the gut microbiome.^41^ To identify a direct relation, further comprehensive research is needed.

In a recent study, Davoodi et al.^42^ observed a positive correlation between asthma and socioeconomic status. In two studies conducted in Ecuador by Jahnke et al.,^15^ the authors noted an increased rate of CS in Ecuadorian women with higher income and suggested that the mode of delivery may be associated with an altered inflammatory response to infection and changes in the proportion of cells involved in the allergic response.^23^ Our study substantiates these conclusions since we were also able to delineate an increased occurrence of allergic diseases in higher social strata. Regarding sex, age, and mode of delivery, socioeconomic status appeared to be a significant predictor of asthma, physician‐diagnosed rhinitis, the occurrence of rhinitis symptoms (such as itching of the nasal tract, nasal secretion, and blocked nose), and food allergies. In a separate analysis, the significant correlation remained within the middle and high socioeconomic classes with regard to physician‐diagnosed asthma, wheezing, and the occurrence of pimples and eczema with itching over 6 months. High socioeconomic status could be considered as meeting the criteria of industrialized countries, but, as previously mentioned, environmental factors such as pollution and early life infection with helminths have been demonstrated to be different in nonindustrialized countries. No significant correlations were found in the low socioeconomic group. However, it must be considered that a majority of children in this group were delivered vaginally and the missing associations can, therefore, be attributed to an imbalance in the collection of data and the low number of respondents in this group. In a recently published comparative analysis of the public and private health systems in Ecuador, the CS rate in the private health system was reported to be double that of the public health system's rate.^22^ This may also explain the results obtained in this study.

## 5 LIMITATIONS

In the aforementioned studies, the authors reported inconsistencies in the literature and particularly pointed out that the observed heterogeneity could be owing to several factors, such as differences in methodology, type of study structure, type of population groups examined (adults vs. children), the use of different definitions for the manifestation of asthma, the effect of bias, and the lack of adequate adaptation of the disturbance variables as well as a limited statistical significance because of small sample sizes. Likewise, it is possible that the relationship between CS and allergies differs in different communities and countries.^36^

A major limitation of the present study was that the number of cases included might have been too small to establish significant relationships. This particularly applies to the population with low socioeconomic status. In this group, only 17 cases of cesarean births could be analyzed, and more than half of these cases were reported to be premature births. This suggests that further studies investigating this population are essential to adequately assess the prevalence of allergic diseases after CS among these children. Other potential confounding factors in the population with low socioeconomic status might have been a lack of access to the healthcare system or a lack of knowledge about allergic diseases might result in undiagnosed allergic disorders. Otherwise, some of the proposed mechanisms might also depend on the type of CS delivery (scheduled vs. emergency), which has not been assessed in this study.

## 6 CONCLUSIONS

Delivery via CS is associated with the development of asthma, eczema, and atopic dermatitis in children aged 3–12 years within the Ecuadorian population. The conclusion that more allergies are seen in industrialized countries fails to explain the considerably higher prevalence of allergic diseases in South American countries as compared with the rest of the world. The observed effect applies to a greater extent to children with a middle to higher socioeconomic background. Further research is needed to investigate the possible pathophysiological etiology underlying this predisposition.

## CONFLICT OF INTERESTS

The authors declare that there are no conflict of interests.

## AUTHOR CONTRIBUTIONS

All authors have made substantial contributions to be mentioned in the study, including: conception, acquisition, and/or analysis and interpretation of data; drafting the study and/or revising it for important intellectual content. All authors have given final approval of the version to be published.

## ETHICS STATEMENT

The study was approved by the ethics committee of University San Francisco de Quito (code 2015‐038T), certified by the Ministry of Public Health from Ecuador following guidelines from the Declaration of Helsinki. All samples were anonymized and no data of the patients were made available. Each subject provided written informed consent before enrollment.
